# Modification of Simple Antenna Pattern Models for Inter-Beam Interference Assessment in Massive Multiple-Input–Multiple-Output Systems †

**DOI:** 10.3390/s23229022

**Published:** 2023-11-07

**Authors:** Jarosław Wojtuń, Cezary Ziółkowski, Jan M. Kelner

**Affiliations:** Institute of Communications Systems, Faculty of Electronics, Military University of Technology, 00-908 Warsaw, Poland; cezary.ziolkowski@wat.edu.pl (C.Z.); jan.kelner@wat.edu.pl (J.M.K.)

**Keywords:** wireless communications, 5G, massive MIMO, antenna beam pattern, inter-beam interference, signal-to-interference ratio (SIR), multi-ellipsoidal propagation model (MPM)

## Abstract

The occurrence of cross-beam interference in the received signal is one of the main problems that limit the possibilities of massive multiple-input–multiple-output technology (massive-MIMO) in fifth-generation (5G) systems. Thus, the evaluation of the level of this interference is one of the most important procedures in the spatial planning of currently wireless networks. We propose a novel modification of simple antenna pattern models, which is based only on changing the directivity of real antenna system patterns. This approach is independent of the antenna system’s type, structure, and analytical description. Based on the developed modification, the original methodology for assessing the signal-to-interference ratio (SIR) from adjacent beams of a common antenna system is presented. The change in the radiation direction and the accompanying change in the complex shape and parameters of the real antenna beam pattern is one of the problems that significantly hinders the evaluation of the analyzed interference. Hence, in the presented methodology, we propose using our modification. In this case, the modification is reduced to a proportional change in the directivity concerning the real antenna system, which results from a change in the beam direction. The simulation studies used a multi-ellipsoidal propagation model and a real massive MIMO antenna pattern description from 3GPP. For the SIR error analysis, the 3GPP pattern is used as a reference. The simulation results show that modifying simple antenna pattern models allows us to obtain an SIR error of no more than 3 dB and 0.1 dB under line-of-sight (LOS) and non-LOS conditions, respectively.

## 1. Introduction

In fifth-generation (5G) New Radio (NR) wireless systems, the use of the millimeter wave range makes it possible to introduce new technologies that significantly contribute to increasing the management efficiency of the available radio resources [1,2,3,4]. Thanks to the beamforming and beam direction control system, the massive multiple-input–multiple-output (MIMO) system is an example of technology that enables the spatial separation of network users using the links of the same gNodeB base station [5,6,7]. However, the practical use of this technology requires solving an additional problem related to the need to assess the limitations of mutual orientations of the individual beams of the antenna system. This is due to the presence of inter-beam interference that degrades the quality of information transmission on individual links. The widespread use of the inter- or co-channel spectral resources by adjacent beams causes inter-beam interference occurrence [8,9]. This phenomenon is the impetus for developing mechanisms and methods of inter-beam interference mitigation, suppression, or cancellation (e.g., [10,11,12]). On the other hand, in 5G and beyond networks, this goal is also achieved by implementing new technologies such as ultra-dense network [13,14], device-to-device [15], full duplex, non-orthogonal multiple access [16,17]. These solutions provide inter-beam interference minimization and are crucial for increasing the coverage and spectral efficiency of the network. Numerous works illustrating empirical [8,18] or simulation studies [13,14,19,20,21,22,23] show the importance of analyzing inter-beam interference in mobile networks.

In inter-beam interference research, the orientation, parameters, and shape of individual antenna beam patterns are of crucial importance. Real antenna patterns or simple models may be used in simulation studies. Currently, to ensure an acceptable level of inter-channel interference, the simulation procedure according to the 3rd Generation Partnership Project (3GPP) standard [24] is widely used. As a result, we can evaluate the required angular separation between the individual antenna beams. However, in this case, the assessment of inter-beam interference is limited only to multi-element array antennas. In the case of other types of multi-beam antennas, such as [25,26,27], this approach will significantly differ from the analysis method with 3GPP. There is also a need to develop a methodology for assessing inter-beam interference that will be independent of the type (design) of the antenna system and will only use knowledge of its parameters and patterns. Additionally, the procedure’s high structural and numerical complexity and the inability to deviate from strictly determined research scenarios make it impossible to assess the level of inter-beam interference under any environmental conditions. The complexity of the analytical description of the antenna patterns and the variability in their parameters that occur with the change in their directions is an additional factor that makes it difficult to assess the required angular separation between the beams.

Simple antenna pattern models (e.g., Gaussian, Cosine, Sinc [28,29]), which primarily consider the influence of the antenna’s main lobe, are used to conduct rough calculations and simulations in which the antenna pattern should be considered. The results of such calculations/simulations are usually used at the initial stage of research or analysis, to estimate the trend of changes in the analyzed parameters and phenomena rather than exact results, e.g., at the initial stage of the spatial design of networks. Then, a statistical approach to model the environment (i.e., buildings, terrain) is used, which also does not produce accurate results typical of the deterministic scenario. The main advantage of using simple models is a shorter computation time. Implementing real or more realistic antenna pattern models requires more time and computational resources. Moreover, for certain issues, e.g., interference assessment, the use of simple models may provide sufficient accuracy for the analysis results.

In this paper, we continue our previous research presented in [21,22,23]. Inter-beam interference evaluations for the sub-6 GHz downlink using the real 3GPP massive MIMO [30] and Gaussian patterns are presented in [21] and [22], respectively. Similar studies for the sub-millimeter-wave downlink and uplink scenarios and real 3GPP antenna patterns are shown in [23]. In these cases, the MPM [31,32] was used for modeling radio channels, and in [22,23], we additionally used the 3GPP channel model [24] to compare the results. The obtained signal-to-interference ratio (SIR) results for the Gaussian and 3GPP patterns showed some discrepancies for line-of-sight (LOS) and non-LOS (NLOS) conditions. It resulted from considering only the main lobe in the simplified pattern model. Under LOS conditions for the 3GPP pattern, the extremes in the SIR graphs versus the beam separation angle occurred for angles that are multiples of the antenna half-power beamwidth (HPBW) for both the MPM and 3GPP models. The results in [14] depict a similar effect. These differences motivated us to analyze other simple pattern models and develop their modifications to obtain a more accurate SIR assessment.

In this paper, we show a modification of simple antenna pattern models. This approach is limited to considering the change in directivity of the real antenna pattern, which occurs with a change in the direction of the main lobe. The proposed solution is independent of the antenna system’s type, structure, and analytical description (e.g., [33,34]) or beamforming technique.

This paper is also devoted to the evaluation methodology of interference between the serving (i.e., useful) and adjacent beams. However, here, the main problem focuses on assessing the possibility of simplifying the complex patterns of real antenna systems [35] using simple models such as the Gaussian, Cosine, and Sinc [28,29]. In the case of real antennas with beam control, both the parameters and shapes of the beams change with the change in their orientation [22,30]. This fact significantly increases the numerical complexity of conducting simulation tests and analytical evaluation of the interference level from the co-channel beams.

This paper shows that the proposed modification of simple models provides consistent results of the interference level assessment in relation to the actual antenna beam pattern. The simplicity of the proposed amendment only considers the change in the antenna beam directivity proportional to the change in this parameter for the real beam. Changing the beam orientation while maintaining its shape significantly simplifies the simulation test procedure and enables the assessment of the influence of its individual elements on the level of interference induced in the receiving antenna. The procedure presented in the paper uses the modification of simple models of antenna beam patterns to analyze the level of interference occurring in radio links with multi-beam antenna systems. This method of evaluating the effects of individual antenna beams on the received serving signal determines the originality and novelty of the developed solution. The main contributions in this paper are as follows:

1. We introduce a novel modification of the simple antenna pattern model, which is based only on the knowledge of the real antenna system pattern and is independent of its type, structure, and analytical description.
2. We evaluate the pattern approximation accuracy of the modified and non-modified models against the 3GPP reference pattern model.
3. From the interference evaluation viewpoint, we proposed assessing the effectiveness of simple pattern models based on SIR errors instead of pattern mapping errors, taking the real antenna pattern (i.e., the 3GPP beam pattern in the case of our studies) as a reference.
4. Based on simulation studies, we perform inter-beam interference analysis of the 5G massive MIMO system for modified and non-modified models under LOS and NLOS conditions.
5. We make a recommendation to use selected modified models for different propagation conditions.
6. We make a recommendation to use selected modified models under different propagation conditions for fast engineering calculations at the initial research stage.

The simulation results show that modifying the simple antenna pattern models allows us to obtain an SIR error of no more than 3 dB and 0.1 dB under LOS and NLOS conditions, respectively. Compared with non-modified models, the SIR error is up to 10 dB or 12 dB for the LOS or NLOS scenarios, respectively. On the other hand, the utilization of the non-modified or modified simple models allows for a significant reduction in computation time compared to real patterns. We show that the generation time of simple patterns is about 300 to 520 times shorter compared to 3GPP. Our research also illustrates that, in assessing interference, the directivity of the main lobe becomes the most important parameter (especially under NLOS conditions) and not the shape of the side lobes.

The paper is structured as follows. A short description of the reference model of the multi-beam antenna system is presented in Section 2. Section 3 shows the simple models of antenna patterns in analytical and graphic form. The modification of simple pattern models in relation to a reference antenna is described in Section 4. In Section 5, the MPM-based interference assessment procedure is shown. This section includes a short survey of the MPM, simulation study assumptions, and the obtained results confirming the developed solution’s correctness. The paper finishes with a summary and final remarks in Section 6.

## 2. 3GPP Reference Model of Antenna Patterns

The assessment of adjacent-beam interference levels in 5G wireless systems is based on the antenna pattern model, which is recommended by the 3GPP. This model describes the transmission properties of an antenna array in the form of an ordered set of radiating elements that form $N_{H}$ rows and $N_{V}$ columns. In this case, the analytical form of the array power radiation pattern in spherical coordinates Ω=(θ,ϕ) is as follows [30]:

(1) $$g_{0}\left(\theta ,\phi \right)=A\left(\theta ,\phi \right)+10\log\left(\frac{1}{K}\sum_{k=1}^{K}{\left|\sum_{m=1}^{N_{H}}\sum_{n=1}^{N_{V}}w_{k,n,m}\cdot v_{n,m}\left(\theta ,\phi \right)\right|}^{2}\right),$$

where A(θ,ϕ) is the power radiation pattern of the antenna array element, K is the number of array beams, $v_{n,m}\left(\theta ,\phi \right)$ are the array of factors related to the position of the radiating elements, and $w_{k,n,m}$ are weighting factors of the individual array coefficients, which depend on the orientation of the antenna array.

The radiation pattern of each element of the antenna array is described by the relationship [30]

(2) $$A\left(\theta ,\phi \right)=G_{\max}-\min\left(-\left(A_{H}\left(\phi \right)+A_{V}\left(\theta \right)\right),G_{0}\right),$$

where $G_{0}=30\;\mathrm{dB},$ $G_{\max}=7.5\;\mathrm{dBi}$ represents the gain of the radiating element and is a constant value, $A_{H}\left(\phi \right)$ and $A_{V}\left(\theta \right)$ are the patterns of the radiating element in the azimuth and elevation planes, respectively, and $G_{0}$ represents the ratio of maximum directivity of the antenna to directivity in a specified rearward direction.

Whereas the characteristics $A_{H}\left(\phi \right)$ and $A_{V}\left(\theta \right)$ are defined in logarithmic measure as follows [30]:

(3) $$A_{H}\left(\phi \right)=-\min\left(12{\left(\frac{\phi }{\phi_{3dB}}\right)}^{2},G_{0}\right),\;A_{V}\left(\theta \right)=-\min\left(12{\left(\frac{\theta -\Theta_{0}}{\theta_{3dB}}\right)}^{2},G_{0}\right),$$

where $\phi_{3\mathrm{dB}}$ and $\theta_{3\mathrm{dB}}$ represent the angular spread of the pattern at the level of −3 dB in the azimuth and elevation planes, respectively, i.e., $A_{H}\left(\phi_{3\mathrm{dB}}/2\right)=-3\;\mathrm{dB}$ and $A_{V}\left(\left(\theta_{3\mathrm{dB}}/2\right)+\Theta_{0}\right)=-3\;\mathrm{dB},$ where $\Theta_{0}=90{}^{\circ}.$ In the presented analysis, the values of $\phi_{3\mathrm{dB}}=80{}^{\circ}$ and $\theta_{3\mathrm{dB}}=65{}^{\circ}$ are adopted based on the recommendation in [30].

The antenna array coefficients $v_{n,m}\left(\theta ,\phi \right)$ are determined based on the following formula [30]

(4) $$v_{n,m}\left(\theta ,\phi \right)=\exp\left(2\pi j\left(\left(n-1\right)\frac{d_{V}}{\lambda }\cos\theta +\left(m-1\right)\frac{d_{H}}{\lambda }\sin\theta \sin\phi \right)\right)$$

and the weighting factors $w_{k,n,m}$ are determined by the expression [30]

(5) $$w_{k,n,m}=\frac{1}{\sqrt{N_{H}N_{V}}}\exp\left(2\pi j\left(\left(n-1\right)\frac{d_{V}}{\lambda }\cos\theta_{k}+\left(m-1\right)\frac{d_{H}}{\lambda }\sin\theta_{k}\sin\phi_{k}\right)\right),$$

where λ is the length of the emitted wave, $d_{V}$ and $d_{H}$ are the distances between the radiating elements of the antenna array in the elevation and azimuth directions, respectively, $\theta_{k}$ and $\phi_{k}$ represent the deflection angles of individual antenna beams relative to the perpendicular direction to the antenna surface in the elevation and azimuth planes, respectively.

**Figure 1:**
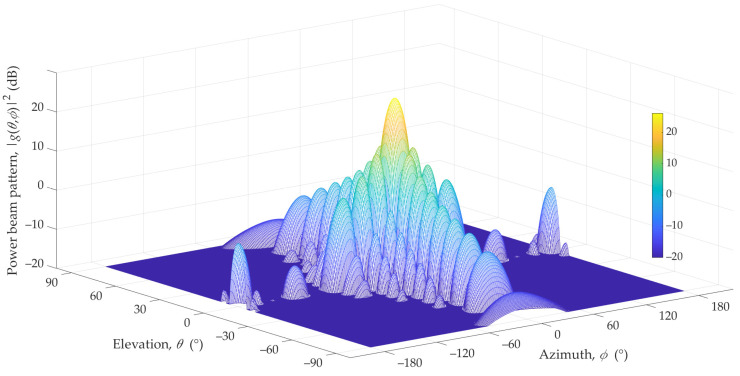
Three-dimensional 3GPP pattern of reference beam [23].

In our analysis, we used a vertical patch as an antenna array of 12 × 8 elements for which the vertical and horizontal spacings are $d_{V}/\lambda =0.7$ and $d_{H}/\lambda =0.5$, respectively. For these parameters, the main lobe HPBWs of the antenna beam are 12.6° and 6° for the azimuth and elevation planes, respectively. Figure 1 depicts the 3D reference pattern model [23].

**Figure 2:**
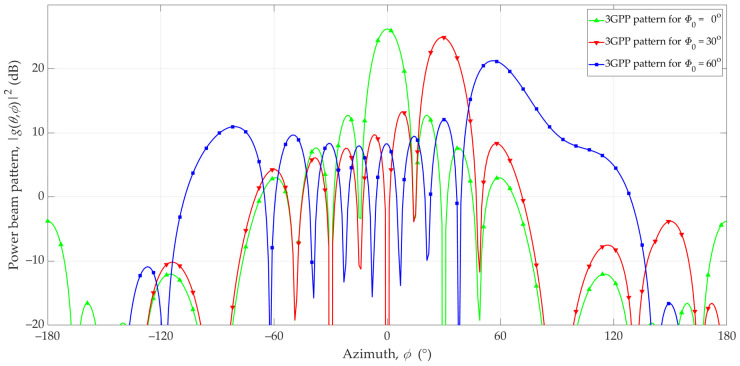
Reference and exemplary 3GPP interfering beams in the azimuth plane.

**Figure 3:**
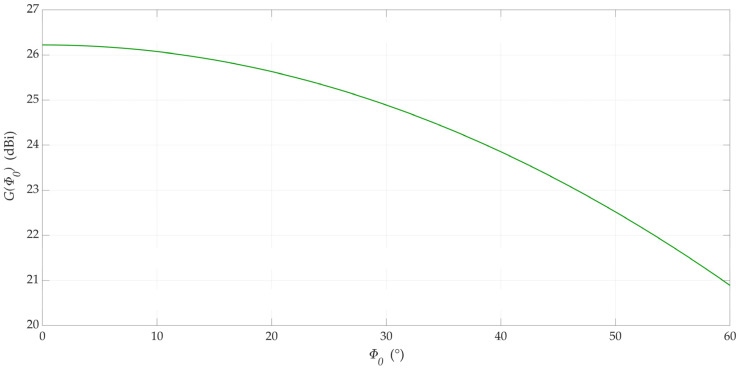
Directivity of 3GPP beams versus maximum beam direction.

Unfortunately, the radiation pattern of the antenna array, which is generated based on Equation (1), changes along with the change in direction of the main lobe. This fact is presented in Figure 2. In the azimuth plane, changes in the direction of the main lobe for different directions Φ_0_ = 0°, Φ_0_ = 30°, and Φ_0_ = 60° are 26.2 dBi, 24.9 dBi, and 20.9 dBi, respectively. Changes in the direction of the main lobe are also accompanied by a change in the directivity of the antenna array. For the analyzed set of structural parameters, the changes in the pattern directivities are shown in Figure 3.

As shown in Figure 2, changing the direction of the beam affects the change in the main lobe level. Figure 3 shows that, for the analyzed antenna patch, the change in directivity can be as much as 5 dB for different Φ_0_ values. This effect should be considered when using simple models of antenna patterns.

## 3. Simple Models of Antenna Patterns

Simple antenna patterns can be modeled by mathematical formulas. Gaussian or circular functions are helpful [28]. To model the antenna power radiation patterns, we adopted the MATLAB function from the Phased Array System Toolbox [29].

The normalized Gaussian pattern is as follows [29]:

(6) $$g\left(\theta ,\phi \right)=\exp\left(-2\ln2{\left(\frac{\phi -\Phi_{0}}{HPBW_{\phi }}\right)}^{2}\right)\exp\left(-2\ln2{\left(\frac{\theta -\Theta_{0}}{HPBW_{\theta }}\right)}^{2}\right).$$

The normalized Cosine pattern is as follows [29]:

(7) $$g\left(\theta ,\phi \right)=\cos^{m}\left(\phi -\Phi_{0}\right)\cos^{n}\left(\theta -\Theta_{0}\right),$$

where

(8) $$m=-\frac{\log_{10}\sqrt{2}}{\log_{10}\left(\cos\left(HPBW_{\phi }/2\right)\right)},n=-\frac{\log_{10}\sqrt{2}}{\log_{10}\left(\cos\left(HPBW_{\theta }/2\right)\right)}.$$

The normalized Sinc pattern is as follows [29]:

(9) $$g\left(n(\theta ),m(\phi )\right)=\frac{\sin(m)}{m}\frac{\sin(n)}{n},$$

where

(10) $$m=\frac{x\sin(\phi -\Phi_{0})}{\sin\left(HPBW_{\phi }/2\right)},n=\frac{x\sin(\theta -\Theta_{0})}{\sin\left(HPBW_{\theta }/2\right)},$$

and *x* is a solution of $\sin(x)=x/\sqrt{2}$.

The numerators above *HPBW_ϕ_* and *HPBW_θ_* represent the HPBWs for the azimuth and elevation planes, respectively. Power pattern is defined as ${\left|g\left(\theta ,\phi \right)\right|}^{2}$.

Moreover, we used the MATLAB function to determine the directivity of antennas [29]. Table 1 depicts the results. Additionally, we show the difference between the directivity of the simple models and 3GPP pattern, Δ*G*.

When modeling 3D patterns and calculating their directivities, the HPBWs of the antennas are considered in both the azimuth and elevation planes. Since, in further considerations, we analyze the change in the beam direction of antenna patterns only in the azimuth plane, this plane will significantly impact the shaping of SIR graphs.

**Figure 4:**
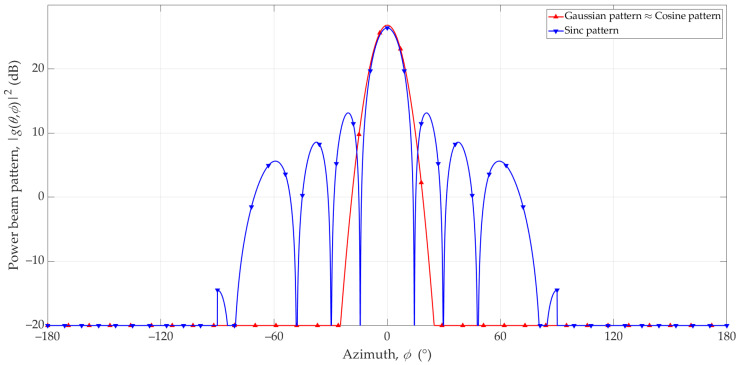
Patterns of basic antenna models in the azimuth plane.

Figure 4 depicts power patterns of simple antennas in dB and additionally considers the directivity of antennas. We also assume ${\left|g\left(\theta ,\phi \right)\right|}^{2}=-20\;\mathrm{dB}$ for all angles (θ,ϕ), where ${\left|g\left(\theta ,\phi \right)\right|}^{2}\leq -20\;\mathrm{dB}$ is based on Equation (6), (7), or (9).

The power patterns for the Gaussian and Cosine models are very similar. To assess the similarity of the two patterns for antenna direction Φ_0_, we determine the root mean square error (RMSE), which is defined as:

(11) $$RMSE\left(\Phi_{0}\right)\;(\mathrm{dB})=10\log_{10}\sqrt{\frac{1}{M}\sum_{i=1}^{M}{\left(g_{1}\left({\left(\theta ,\phi \right)}_{i},\Phi_{0}\right)-g_{2}\left({\left(\theta ,\phi \right)}_{i},\Phi_{0}\right)\right)}^{2}},$$

where $g_{1}\left({\left(\theta ,\phi \right)}_{i},\Phi_{0}\right)$ and $g_{2}\left({\left(\theta ,\phi \right)}_{i},\Phi_{0}\right)$ mean two analyzed patterns, *M* is a set of ${\left(\theta ,\phi \right)}_{i}$ elements.

For the analyzed case, $g_{1}\left(\left(\theta ,\phi \right),\Phi_{0}\right)$ and $g_{2}\left(\left(\theta ,\phi \right),\Phi_{0}\right)$ are the Gaussian and Cosine patterns, respectively. Regardless of the choice of Φ_0_, the RMSE equals −8.4 dB. Due to this relatively small error value, we limited the further analyses to the Gaussian and Sinc models.

Real antenna patterns are used at further research stages, which are based on simulations for deterministic scenarios. At the initial research stages, e.g., for the spatial design of mobile networks using a statistical approach, simple models are often used. Their main advantage is a shorter computation time and lower requirements for computational resources. In Table 2, the mean times for determining antenna patterns in relation to calculations for 1000 generated patterns with an angular resolution of 0.1° are presented.

The presented times are small for all models, but calculations or simulations at the initial stage of research are often performed on millions of statistically independent cases. Referring to Table 2, the computation time needed to generate a non-modified simple model is, on average, 320 (Sinc) to 520 (Gaussian) times shorter than for the real 3GPP model. The developed modification does not add significant computational costs, i.e., the modified simple models use an approximately 300–470 times shorter execution time compared to the 3GPP pattern.

Our simulations were performed in the MATLAB (R2021b) environment on the Dell (Round Rock, TX, USA) computer with an Intel(R) Core(TM) i7-4790K CPU @ 4.00 GHz processor, 32 GB random access memory (RAM), and Windows 10 Education (22H2) operating system.

## 4. Modification of Simple Models

**Figure 5:**
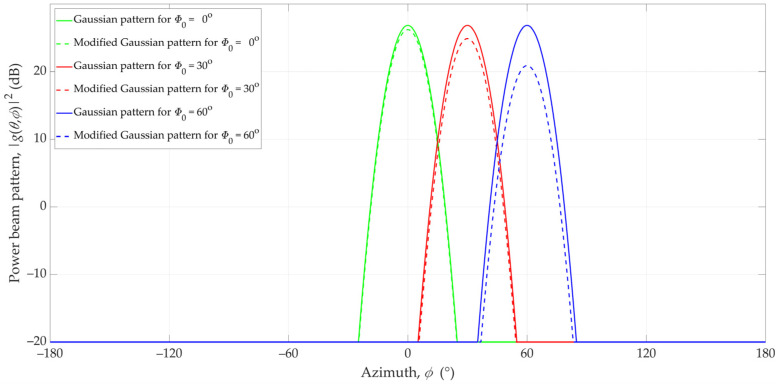
Patterns of non-modified and modified Gaussian antennas in the azimuth plane for different directions (Φ_0_ = 0°, 30°, and 60°).

**Figure 6:**
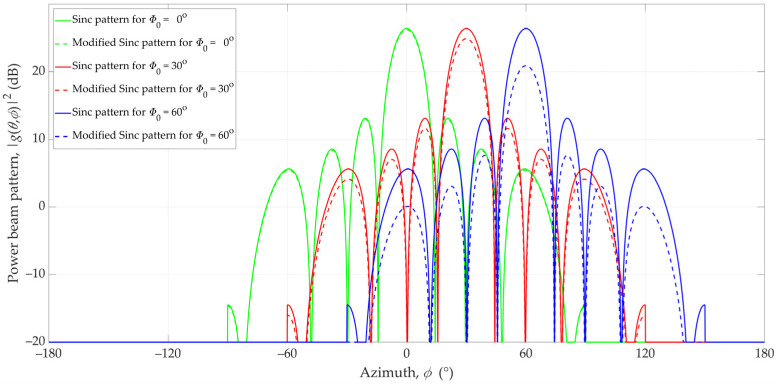
Patterns of non-modified and modified Sinc antennas in the azimuth plane for different directions (Φ_0_ = 0°, 30°, and 60°).

Considering the directivity of the 3GPP antenna pattern (see Figure 3), we modified the Gaussian and Sinc pattern models. The beam patterns for the non-modified and modified Gaussian and Sinc antenna for directions Φ_0_ = 0°, Φ_0_ = 30°, and Φ_0_ = 60° in the azimuth plane are illustrated in Figure 5 and Figure 6, respectively.

**Figure 7:**
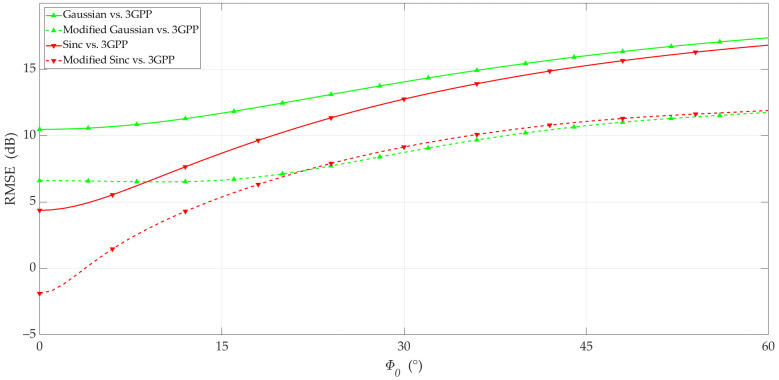
RMSE between 3GPP and Gaussian or Sinc antenna patterns versus Φ_0_.

To assess the difference between the 3GPP and Gaussian and Sinc (non-modified and modified) antennas, we calculated the RMSE based on Equation (11). For the analyzed case, $g_{1}\left(\left(\theta ,\phi \right),\Phi_{0}\right)$ and $g_{2}\left(\left(\theta ,\phi \right),\Phi_{0}\right)$ are the 3GPP pattern and non-modified or modified models. The results are depicted in Figure 7.

The graphs in Figure 7 show that the proposed modification of the simple models al-lows for a reduction in the RMSE by about 5 dB compared to the non-modified models. We can see that the difference in RMSE between the same type of non-modified and modified pattern models for small values of the beam directions (i.e., scanning angles). The RMSE differences were about 4 dB or 6.5 dB for the Gaussian and Sinc, respectively. These differences resulted from a slight difference (Δ*G*) in directivity between the non-modified models and the 3GPP pattern for Φ_0_ = 0° (see Table 1). The values of Δ*G* are considered in the modified models.

In our opinion, analyzing the pattern mapping error (i.e., RMSE) is not the best approach when evaluating simple models against real ones. From the interference evaluation viewpoint, we proposed assessing the effectiveness of simple pattern models based on SIR errors, which is presented in the remainder of this paper. On the other hand, slight differences in the directivity of the patterns caused significant changes in the RMSE and SIR error. This clearly shows a crucial role of this parameter, which we used in our modification.

## 5. MPM-Based Interference Assessment

### 5.1. MPM

Assessing the inter-beam interference approximation accuracy by selected antenna pattern models requires considering the propagation environment’s transmission properties. This is ensured using geometrical modeling of propagation phenomena in simulation studies. In this case, a geometric structure is a semi-ellipsoid set representing the scattering elements’ potential locations. A power delay profile (PDP) is the basis for constructing this geometric structure, which maps the propagation conditions of electromagnetic waves in a real environment. In the PDP, the occurrence of local extremes shows that the received signal can be treated as a superposition of components that reach the receiver (Rx) from different directions. The lengths of the propagation paths of all the components that form the individual extremes are the same. This is due to the same propagation time (i.e., delay). Thus, for individual delays, the potential locations of the scattering elements, from which the signal components reach the Rx, are determined by the surfaces of the semi-ellipsoids. Hence, this propagation model is called the MPM. The focal points of individual half-ellipsoids are determined by the locations of the transmitter (Tx) and Rx. The major $a_{n},$ and minor $b_{n},$ $c_{n},$ axes corresponding to the *n*th delays $\tau_{n}$ are described by the dependencies:

(12) $$a_{n}=\frac{1}{2}\left(c\tau_{n}+D\right),b_{n}=c_{n}=\frac{1}{2}\sqrt{c\tau_{n}\left(c\tau_{n}+2D\right)},$$

where *c* denotes the speed of light and *D* is the Tx–Rx distance.

**Figure 8:**
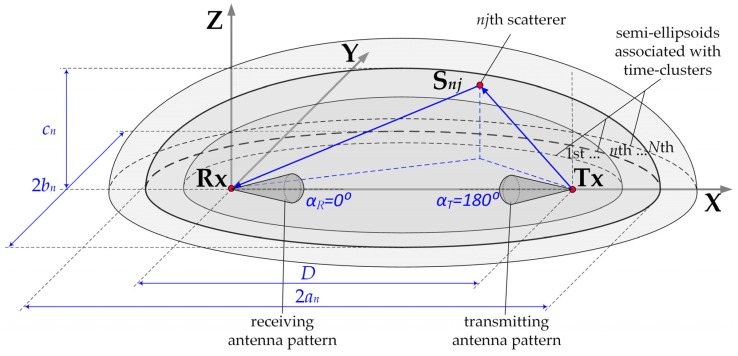
Spatial structure of MPM [22].

The spatial structure of the MPM is shown in Figure 8.

Using the geometric model makes it possible to assess the interference between spatially differentiated channels, which are constructed based on multi-beam antenna systems. The MPM considers the influence of the spatial location of antenna beams in shaping the power distribution of the electromagnetic field that occurs in the vicinity of the receiving antenna. To determine this distribution, a similar method to the ray-tracing method is used. However, in this case, we did use not rays but radio wave propagation paths that represent the directions of energy flow of the transmitted signal. In the simulation process, the intensity of path generation in particular directions is proportional to the power radiation pattern of the transmitting antenna. In contrast, the intensity of the paths that arrive from the scattering elements $S_{n}\;\left(\forall n=1,2,\ldots ,N\right)$ to the Rx, represents the power angular spectrum (PAS), $p_{0}\left(\theta ,\phi \right),$ around the receiving antenna. A detailed description of the PAS determination procedure that uses the MPM is included in [31,32]. The product of $p_{0}\left(\theta ,\phi \right)$ and the antenna radiation pattern can be interpreted as the angular distribution of the signal power p(θ,ϕ) that is “seen” at the output of the receiving antenna. When the radiation direction of the transmitting antenna is equal to $\Phi_{0},$ then this distribution is the basis for determining the power $P(\Phi_{0})$ of the received signal in accordance with the relationship:

(13) $$P\left(\Phi_{0}\right)=\frac{1}{4\pi }\iint_{\Omega }p\left(\left(\theta ,\phi \right),\Phi_{0}\right)\sin\theta d\theta d\phi .$$

The relationship described by Equation (13) is the basis for determining both the power $P_{S}(\Phi_{0S})$ of the serving signal and the power $P_{I}(\Phi_{0I})$ of the interfering signal, which come from the main lobes in directions $\Phi_{0S}$ and $\Phi_{0I},$ respectively. Determining these powers makes it possible to evaluate the interference level as a function of the angular separation between the beams of the antenna system.

### 5.2. Simulation Study Assumptions

The simulation tests aimed to assess the possibility of simplifying the complex patterns of real antenna systems [33] by using simple models such as the Gaussian or Sinc patterns. The studies focused on determining the SIR relationship with the separation angle Δα. The simulations were carried out for the distance *D* = 100 m between the Tx (gNodeB) and Rx (UE). The carrier frequency was fixed at 28 GHz, typical for the 5G micro- and pico-cells, where multiple sub-arrays and beamforming technologies are planned for implementation.

We adopted the 3GPP recommendations [30], and non-modified or modified simple antenna patterns to model the Tx antennas. The Rx antenna consists of a single element with a main lobe HPBW of about 90°.

In our scenario, we assumed that the Tx was generating two beams (serving and interfering) in the selected sector that were operating in the same sub-band (frequency channel). Thus, the SIR assessment comes down to determining the $P_{S}$ and $P_{I}$ powers induced in the Rx antenna that come from the signals generated by the serving and interfering beams, respectively. The SIR definition based on the PAS for two beams is as follows:

(14) $$\begin{array}{l} SIR(\Delta \alpha )\left(\frac{W}{W}\right)=\frac{P_{s}(\Phi_{0S}=0{}^{\circ})}{P_{I}(\Phi_{0I}=\Delta \alpha )}\\ SIR(\Delta \alpha )\;(\mathrm{dB})=P_{s}\;(\Phi_{0S}=0{}^{\circ})\;(\mathrm{dBm})-P_{I}(\Phi_{0I}=\Delta \alpha )\;(\mathrm{dBm}) \end{array}$$

To assess the accuracy of the SIR approximation, we used the measure ΔSIR defined as:

(15) $$\Delta SIR(\Delta \alpha )\;(\mathrm{dB})=\left|10\log_{10}\left(\frac{SIR_{Model}(\Delta \alpha )\;\left(W/W\right)}{SIR_{3GPP}(\Delta \alpha )\;\left(W/W\right)}\right)\right|$$

**Figure 9:**
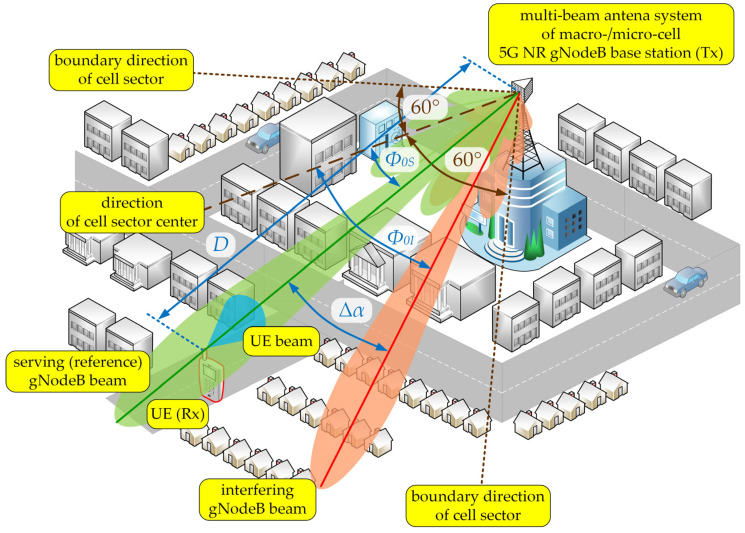
Spatial scenario for downlink simulation studies [22].

The serving (reference) Tx and Rx beams were aligned, i.e., directed to each other ($\alpha_{T}=0{}^{\circ}$ and $\alpha_{R}=180{}^{\circ}$, see Figure 8). In relation to the direction of the cell sector center, the reference and interfering Tx beams are oriented in $\Phi_{0S}$ and $\Phi_{0I}$ directions, respectively (see Figure 9). Thus, the separation angle of the beams is defined as:

(16) $$\Delta \alpha =\Phi_{0S}-\Phi_{0I}$$

Then, the interfering beam orientation in relation to the Tx-Rx direction was equal to Δα. In our tests, the direction of the reference Tx beam overlapped with the cell sector center, i.e., $\Phi_{0S}=0{}^{\circ}.$ Hence, we considered the change in separation angle in the ranges of 0°÷60° which corresponds to half of a 120° sector. The tests were carried out for LOS and NLOS conditions.

In the simulation studies, the following assumptions were made:

- An illustrative spatial scenario, as shown in Figure 9;
- Carrier frequency is equal to $f_{c}=28\;\mathrm{GHz};$
- PDPs are based on tapped-delay line (TDL) models from the 3GPP TR 38.901 standard [24], i.e., the TDL-B and TDL-D for NLOS and LOS conditions, respectively; these TDLs are adopted for analysis and RMS delay spread, $\sigma_{\tau },$ for so-called the normal-delay profile and urban macro (UMa) scenario, i.e., $\sigma_{\tau }=266\;\mathrm{ns};$
- The Rician factor defining the direct path component in the scenario for LOS conditions is appropriate for TDL-D [24], i.e., κ=13.3 dB;
- The intensity coefficients of the local scattering components, i.e., the 2D von Mises distribution parameters, are equal to γ=60;
- The distance between the TX and RX is equal to *D* = 100 m;
- The range of changes in the beam separation angle is [0°, 60°] with step size of 0.1° in the azimuth plane.

### 5.3. Simulation Results

**Figure 10:**
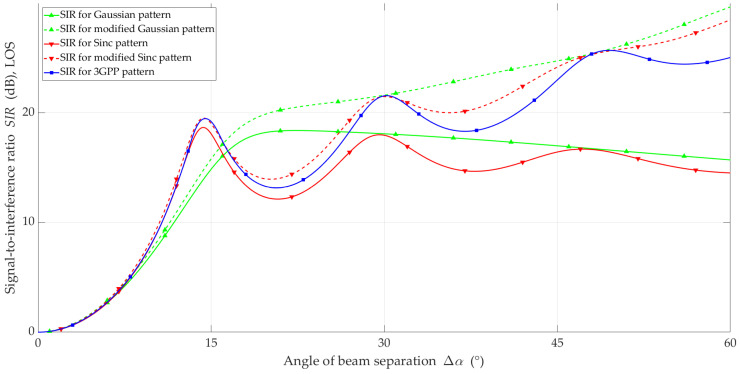
*SIR* versus beam separation angle under LOS conditions.

**Figure 11:**
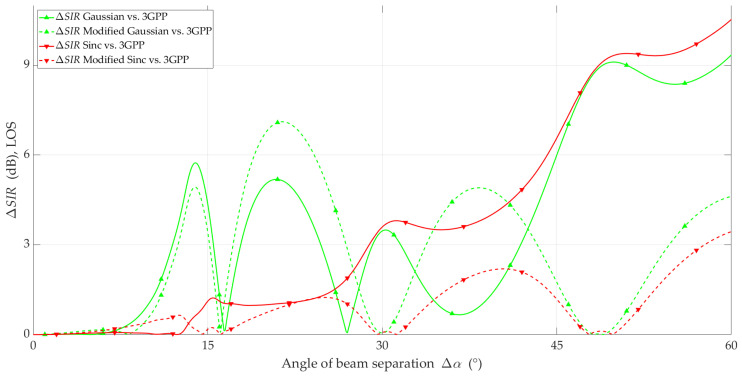
Δ*SIR* versus beam separation angle under LOS conditions.

**Figure 12:**
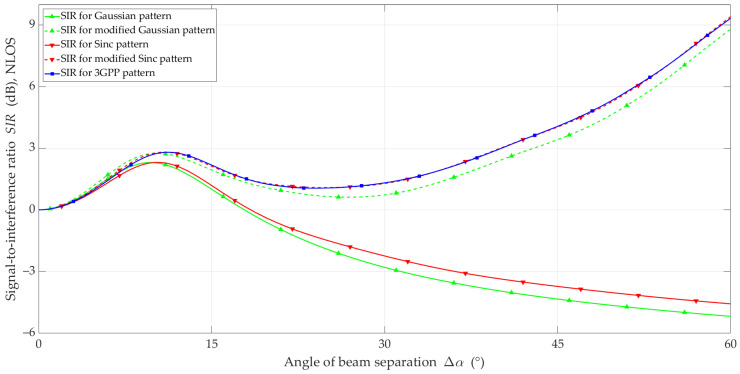
*SIR* versus beam separation angle under NLOS conditions.

**Figure 13:**
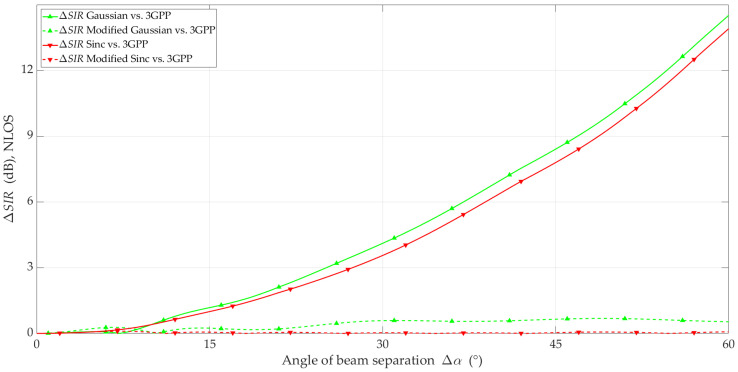
Δ*SIR* versus beam separation angle under NLOS conditions.

The simulation studies were carried out using the MPM for the analyzed scenario and the adopted assumptions. The simulation results in the form of *SIR* and ΔSIR versus beam separation angle are illustrated in Figure 10, Figure 11, Figure 12 and Figure 13 for LOS and NLOS conditions, respectively.

In general, under LOS conditions, for all analyzed models, the SIR increased with the increase in the beam separation angle. Characteristic local maxima occur for the 3GPP reference and Sinc models. This is because the side lobes were included in the antenna pattern. Their location is related to the HPBW designated for the main lobe. The lack of side lobes in the Gaussian model significantly differentiates the nature of the obtained SIR curves. The proposed modification of the simple models ensures that the upward trend of *SIR* is maintained with the increase in Δα, similar to 3GPP. In the case of the non-modified simple models, for Δα>15°, we observed a downward trend.

In Figure 11, the ΔSIR of the four analyzed models under LOS conditions are shown. Based on this, we can conclude that the modified Sinc model provides the most accurate representation of SIR relative to the 3GPP reference model for all analyzed values of Δα. This fact is evident because the Sinc model (see Figure 6) provides an additional opportunity to reproduce the influence of side lobes.

The advantage of the developed solution is evident in the NLOS scenario. In this case, the change in the *SIR* versus Δα curve for the modified models is very similar to the curve for the reference model. Similar to the LOS conditions, the modification of the models produced an upward *SIR* trend for Δα>25°. It should also be noted that the SIR curves for the 3GPP and modified Sinc patterns coincide over the entire range of beam separation angles.

Figure 13 depicts ΔSIR versus Δα for the four analyzed models under NLOS conditions. Compared to the LOS conditions, we can observe the clear effectiveness of the proposed modification. In the entire range of analyzed angles, the ΔSIR error did not exceed 0.7 dB for the modified Gaussian, and for the modified Sinc, the maximum ΔSIR error was no more than 0.1 dB.

The RMSE and mean error (ME) metrics were the basis for the quantitative assessment of the comparative analyses. These metrics are determined by the expressions:

(17) $$RMSE\;(\mathrm{dB})=10\log_{10}\sqrt{\frac{1}{N}\sum_{i=1}^{N}{\left(SIR_{Model}\left(\Delta \alpha_{i}\right)-SIR_{3GPP}\left(\Delta \alpha_{i}\right)\right)}^{2}},$$

(18) $$ME\;(\mathrm{dB})=10\log_{10}\left(\frac{1}{N}\sum_{i=1}^{N}\left|SIR_{Model}\left(\Delta \alpha_{i}\right)-SIR_{3GPP}\left(\Delta \alpha_{i}\right)\right|\right),$$

where $\Delta \alpha_{1}=0{}^{\circ},\;\Delta \alpha_{2}=0.1{}^{\circ},\;\ldots ,\;\Delta \alpha_{N}=60{}^{\circ},$ i.e., N=601.

The results are shown in Table 3.

The analysis of the obtained results shows that the modified Sinc provides the most accurate representation of the 3GPP antenna pattern in the interference analysis under both LOS and NLOS conditions. In the NLOS scenarios, using the modified Gaussian modeling, only the antenna’s main lobe may be sufficient to assess interference. This fact results from the small influence of the pattern’s side lobes on the received power, which is related to the occurrence of multipath propagation and the lack of a direct path.

## 6. Summary

In this paper, we presented a new approach to interference analysis. The high complexity level of the analytical description of the real multi-antenna pattern significantly hinders the inter-beam interference evaluation. The simple antenna pattern models provide the opportunity to simplify this analysis. However, these models introduce errors in estimating the SIR. For this reason, we proposed modifying the simple antenna models, which provides a compromise between the SIR assessment accuracy and the complexity of the antenna pattern description. The developed modification considers the change in directivity of the real 3GPP antenna pattern along with the change in its main lobe direction.

The simulation studies using the MPM for selected spatial scenarios under LOS and NLOS conditions were performed to check the SIR estimation accuracy. Our tests considered the Gaussian, Cosine, and Sinc models and their modified versions. For the scenario and propagation conditions, we determined *SIR* and ΔSIR as a function of the separation angle between the serving and interference beams. ΔSIR, as an accuracy measure, is the SIR ratio between the analyzed modified or non-modified simple and 3GPP reference pattern models. Then, based on these values, the RMSE and ME were determined. The simulation results show that the proposed modification better reflects the SIR changes for the 3GPP pattern compared to the non-modified Gaussian and Sinc models. It follows that the modified Sinc provides the smallest approximation error under LOS and NLOS conditions. However, for NLOS conditions, the modified Gaussian, which has the most straightforward analytical formula, can also be used.

## Figures and Tables

**Table 1:** Directivity of antenna for Φ_0_ = 0°.

Antenna Pattern	*G* (dBi)	Δ*G* (dB)
Gaussian	26.83	0.61
Cosine	26.84	0.62
Sinc	26.40	0.18
3GPP	26.22	0.00

**Table 2:** Mean time for determining antenna patterns.

Antenna Pattern	Mean Execution Time for Non-Modified Models (ms)	Mean Execution Time for Modified Models (ms)
Gaussian	0.179	0.196
Cosine	0.252	0.266
Sinc	0.291	0.304
3GPP	92.935	-

**Table 3:** RMSE and ME for SIR.

Comparison of SIR	Condition	RMSE (dB)	ME (dB)
Gaussian vs. 3GPP	LOS	21.41	19.46
Sinc vs. 3GPP		21.51	19.46
Modified Gaussian vs. 3GPP		22.06	19.71
Modified Sinc vs. 3GPP		19.63	16.50
Gaussian vs. 3GPP	NLOS	3.95	2.07
Sinc vs. 3GPP		3.89	1.95
Modified Gaussian vs. 3GPP		−4.58	−6.02
Modified Sinc vs. 3GPP		−14.95	−16.82

## Data Availability

The data presented in this study are available on request from the corresponding author.
